# Large scale patterns of genetic variation and differentiation in sugar maple from tropical Central America to temperate North America

**DOI:** 10.1186/s12862-015-0518-7

**Published:** 2015-11-19

**Authors:** Yalma L. Vargas-Rodriguez, William J. Platt, Lowell E. Urbatsch, David W. Foltz

**Affiliations:** Department of Biological Sciences, Louisiana State University, Baton Rouge, Louisiana 70803 USA

**Keywords:** *Acer saccharum*, Cloud forest, Divergence time, Gene flow, Genetic diversity, Last glacial maximum, Microsatellite, Phylogeography, Pleistocene, Pliocene, Range edge

## Abstract

**Background:**

Geological events in the latter Cenozoic have influenced the distribution, abundance and genetic structure of tree populations in temperate and tropical North America. The biogeographical history of temperate vegetation that spans large ranges of latitude is complex, involving multiple latitudinal shifts that might have occurred via different migration routes. We determined the regional structuring of genetic variation of sugar maple (*Acer saccharum* subsp. *saccharum*) and its only subspecies in tropical America (*Acer saccharum* subsp. *skutchii*) using nuclear and chloroplast data. The studied populations span a geographic range from Maine, USA (46°N), to El Progreso, Guatemala (15°N). We examined genetic subdivisions, explored the locations of ancestral haplotypes, analyzed genetic data to explore the presence of a single or multiple glacial refugia, and tested whether genetic lineages are temporally consistent with a Pleistocene or older divergence.

**Results:**

Nuclear and chloroplast data indicated that populations in midwestern USA and western Mexico were highly differentiated from populations in the rest of the sites. The time of the most recent common ancestor of the western Mexico haplotype lineage was dated to the Pliocene (5.9 Ma, 95 % HPD: 4.3–7.3 Ma). Splits during the Pleistocene separated the rest of the phylogroups. The most frequent and widespread haplotype occurred in half of the sites (Guatemala, eastern Mexico, southeastern USA, and Ohio). Our data also suggested that multiple Pleistocene refugia (tropics-southeastern USA, midwestern, and northeastern USA), but not western Mexico (Jalisco), contributed to post-glacial northward expansion of ranges. Current southern Mexican and Guatemalan populations have reduced population sizes, genetic bottlenecks and tend toward homozygosity, as indicated using nuclear and chloroplast markers.

**Conclusions:**

The divergence of western Mexican populations from the rest of the sugar maples likely resulted from orographic and volcanic barriers to gene flow. Past connectivity among populations in the southeastern USA and eastern Mexico and Guatemala possible occurred through gene flow during the Pleistocene. The time to the most common ancestor values revealed that populations from the Midwest and Northeast USA represented different haplotype lineages, indicating major divergence of haplotypes lineages before the Last Glacial Maximum and suggesting the existence of multiple glacial refugia.

**Electronic supplementary material:**

The online version of this article (doi:10.1186/s12862-015-0518-7) contains supplementary material, which is available to authorized users.

## Background

Geological events have affected the distributions and levels of gene flow among populations of North American tree species. Repeated glacial and interglacial periods promoted range shifts of a number of tree species during late Pliocene and the Quaternary [1–4]. Studies indicate different genetic responses to range contraction and expansion [5, 6]. For instance, patterns of genetic diversity in temperate North American trees (e.g., *Carya illinoinensis, Liquidambar styraciflua*, *Fagus grandifolia*) include higher diversity in southern, never-glaciated areas, and lower diversity in northern areas where glaciated landmasses existed [7–11]. Furthermore, there is evidence of the impact of the Quaternary glaciations on the population genetic structure of species (e.g., *Acer rubrum, A. saccharinum, Quercus rubra*) that survived in multiple refugia closer to the ice margin [10–12]. Populations of temperate tree species in the subtropics (e.g., *Pinus chiapensis, Picea chihuahuana, Pseudotsuga menziesii*, *Fagus grandifolia* var. *mexicana*) also show genetic differentiation related to the history of migration and isolation during glacial and interglacial periods [13–16]. Additionally, the distributions of many North American temperate species include disjunct populations in cloud forests in Mexico and Central America. These isolated populations may have originated by ancient vicariant events or through range fluctuations. These fluctuations may have involved expansions into low elevation areas at low latitudes during glacial episodes, and contractions to high elevation refuges during warmer interglacial periods [17, 18].

Periods of connectivity and disjunction among North American and Central American refugial areas and their consequences on the genetic structure of the temperate tree populations are not known for most species. Limited geographical sampling reduces the capacity to reconstruct historical refugia and identify the spatial location of genetic breaks in temperate tree species at a continental level. Assessing large-scale relationships of populations should elucidate the importance of range expansions and contractions in producing the current distribution patterns of species, as well as generate hypotheses regarding the genetic structure and diversity of temperate tree lineages affected by glaciation in the latter part of the Cenozoic.

*Acer saccharum* (sugar maple) is a widespread temperate tree species. Study of the genetics of this species has the potential to elucidate a late Pliocene and Quaternary history that may be shared by other elements of the North America flora. The species has a continuous distribution from southern Quebec to the southeastern USA, and then is disjunct in distribution between the eastern USA, Mexico, and Guatemala [19]. Fossil pollen data suggest that *A. saccharum* underwent northward geographic expansions from a single continuous ice-free refugium in the Southern USA about 21,000 years B.P., around the beginning of the retreat of the Laurentide ice sheet [17]. The evidence of the existence and location of glacial refuges of sugar maple farther north than the Lower Mississippi Valley has been inconclusive [20]. Nonetheless, the pollen record of some temperate eastern taxa suggests the presence of small populations in the upper Midwest and in the Appalachian region during glaciations [21, 22]. In addition, according to genetic data, *Acer rubrum* likely existed north of their Pleistocene pollen-based range limits, but, it is not yet known if the same applies to *A. saccharum* [21]. *Acer saccharum* exhibits some genetic differentiation in southeastern Canada and northeastern USA, but less genetic variation than other temperate trees or shrubs [23]. In addition, some sugar maple populations in Canada possess limited genetic differentiation, possibly due to common ancestry or recent colonization after a glacial period [24, 25]. These genetic studies of sugar maple in North America were conducted near its northern range limit, preventing analysis of range expansions as a result of late Pliocene and Quaternary events. Furthermore, southern sugar maple populations with disjunct distributions from northern Mexico to Guatemala have not been considered in reconstructions of historical migration corridors.

We investigated patterns of genetic variation and structure in extant populations of sugar maples in the tropics and explored their genetic relationships with populations in the temperate United States. We used these data to infer their evolutionary history and large-scale connectivity. We quantified within- and among-population genetic variation across the species range using chloroplast sequences and microsatellite loci. We hypothesized that ancestral haplotypes have persisted in tropical populations in unglaciated areas that have changed little over long periods, and that these populations have harbored higher genetic diversity. Next, we estimated divergence times of haplotypes among populations and considered whether observed genetic subdivisions were temporally consistent with the late Pliocene and Quaternary geologic events. Finally, we tested the hypotheses of expanding/contracting populations and the presence of a single refugium versus multiple refugia during episodes of glaciation. Based on our study, we propose the location of more than one continuous ice-age refugium in North America, as well as potential migration routes in relation to the complex phylogeographical patterns of North and Central American hardwood forests.

## Methods

### Field and laboratory procedures

#### Sampling and DNA extraction

Sampling of *Acer saccharum* subsp. *saccharum* was conducted in old-growth forests of USA. Sites where the species identity was doubted by botanists were not sampled. Sampling in Mexico and Guatemala included all known populations where *A. saccharum* subsp. *skutchii* has been recorded [19]. These sugar maples belong to the series *Saccharodendron* (Rafinesque) Murray, section *Acer*, characterized by apetalous flowers and connate calyces. *Acer saccharum* subsp. *skutchii* represents the only member of the series distributed in Mexico and Guatemala [26]. Both subspecies are morphologically the most similar of the series [26].

**Fig. 1:**
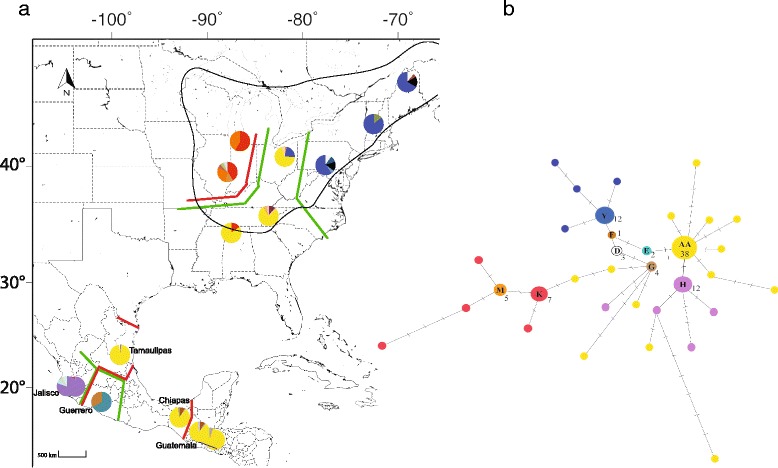
Map of collection sites and haplotype network in sugar maples. **a** Distribution of chloroplast haplotypes observed. Pie charts indicate the frequency of haplotypes within each population. Each circle corresponds to a locality. Haplotypes found are indicated by different colors. Barrier boundaries using nuclear data are red lines; boundaries based on chloroplast data are green lines. Boundaries, calculated in Barrier v.2.2 using the *D*est differentiation estimator for nuclear microsatellites (bootstrap support is 58 % for Midwest, 45 % for West Mexico, 41 % for South Mexico) and the Tamura-Nei population distance measure *D*_A_ for chloroplast haplotypes (bootstrap support is 55 % for Midwest, 50 % for South Mexico, 50 % for West Mexico, 46 % for Northeast). Black, continuous line indicates natural distribution of *A. saccharum* in North America. All know populations in Mexico and Guatemala of *A. saccharum* subsp. *skutchii* are represented in the map. **b** Network of 34 chloroplast DNA haplotypes observed. The identification letter of each haplotype is presented. The size of the circle and the number indicate the observed frequency. The colors correspond to the alleles depicted in the map a)

The sampled populations of sugar maple spanned a geographic range from Maine, USA (46°N), to El Progreso, Guatemala (15°N). Fresh foliage was sampled from 233 adult individuals from 16 native populations (Fig. 1, Additional file 1). Twenty-five to 41 adult individuals (>40 cm diameter at breast height) per population, spaced a minimum of 50 m apart, were sampled in the USA and, where the density permitted, in Mexico. Because of low tree densities in Guatemala, all mature trees observed were sampled regardless of distances among them. The leaves were collected with permits granted by forest reserves. The first author performed the species identification. Leaves were immediately dried in silica gel. Dry leaf material (20–50 mg) was ground using a Mini Beadbeater 8 (BioSpec Products, Bartlesville, OK). The genomic DNA was extracted and purified using a DNeasy Plant Mini Kit (Qiagen, Valencia, CA).

#### Genotyping microsatellites

Six microsatellite loci were amplified using primer pairs and polymerase chain reaction (PCR), the protocol described by Pandey *et al.* [27]. Six out of eight primers had been developed for *Acer pseudoplatanus* and showed high degree of cross-species amplification (MAP9, MAP10, MAP33, MAP34, MAP40, and MAP46). The forward primer was fluorescently labeled using 6-FAM or HEX for MAP10 (Applied Biosystems, Foster City, CA, USA) on the 5- end for detection on an ABI 3130XL (Applied Biosystems, USA).

PCR and fragment analysis were carried out as follows. PCR amplifications of 20 μL aliquots were performed using 20 ng of genomic DNA (quantified with a NanoDrop 2000, Thermo Scientific, Waltham, Massachusetts, USA), 0.2 mm dNTP, 10 μm of each primer, 1.5 mm MgCl_2_, and 1 U HotStarTaq polymerase (HotStarTaq Master Mix, Qiagen, USA). PCR programs consisted of an initial denaturation at 95 °C for 15 min, followed by 30 cycles of 45 s at 94 °C, 45 s at appropriate annealing temperature, and 45 s at 72 °C, and a final extension of 15 min at 72 °C. Annealing temperatures were determined using a gradient PCR and were as follows: 52 °C (MAP9 and MAP33), 55 °C (MAP10), 55.8 °C (MAP34), 58 °C (MAP40), 48 °C (MAP46). Two microliters of PCR product were added to 10.8 μL of Hi-Di Formamide (Applied Biosystems, USA) and 0.2 μL ROX 400 HD size standard and run on an ABI 3130XL automated sequencer. The allele size calling (size of fragments using decimal numbers) was done using GeneMapper v4.1 (Applied Biosystems, USA), and the binning (conversion of alleles into discrete units) was done with Autobin [28]. MICRO-CHECKER v2.2.3 was used to identify genotyping errors such as large allele dropout, stutter peaks, or null alleles [29].

#### Chloroplast DNA sequencing

The *ndhF-rpl32R* intergenic spacer in the small single-copy region and *psbJ-petA* intergenic spacer in the large single-copy region from the chloroplast were amplified [30]. Both regions are noted as highly variable and several haplotypes have been observed [30]. PCR was used to amplify the two regions following the protocol described in Shaw *et al.* [30] with some modifications. Each reaction (25 μL) consisted of 20 ng of genomic DNA, 10 μm of each primer, 0.2 mm dNTP, 1.5 mm MgCl_2_, and 1 U HotStarTaq polymerase (HotStarTaq Master Mix, Qiagen, USA). PCR programs consisted of an initial denaturation at 95 °C for 15 min, followed by 35 cycles of 1 min at 95 °C, annealing at 52 °C for 1 min, followed by a ramp of 0.3 °C/s to 65 °C, and 4 min at 65 °C, and a final extension of 10 min at 66 °C. PCR products were purified using the solid phase reversible immobilization technique and sequenced in both directions using BigDye Terminator v3.1 (Applied Biosystems, USA) on an ABI-PRISM 3730XL. Sequence chromatograms were visually inspected and edited in Sequencher v4.1 (Gene Codes Corp., Ann Arbor, MI, USA), alignment was done in MUSCLE [31] using the European Bioinformatics Institute web platform (http://www.ebi.ac.uk), and manually edited in McClade v4.08 [32] as needed. The sequences have been submitted to GenBank with the accession numbers KT933356–KT933397.

### Data analyses

#### Nuclear microsatellite diversity

Deviations from Hardy-Weinberg Equilibrium (HWE) and linkage disequilibrium were assessed in GENEPOP v4.1 [33]. The test was run using Markov chain parameters of 1000 batches and 10,000 iterations-per-batch. A sequential Bonferroni correction was applied to reduce error rates. The mean number of alleles per locus and per site and the expected (*H*_*E*_) and observed heterozygosity (*H*_*O*_) per locus, as well as the private allelic richness of each population were calculated in the R package “adegenet” [34]. The inbreeding coefficient (*F*_IS_) was estimated for each population, and differences among populations were compared using Mann-Whitney *U*-tests.

Each population was evaluated for evidence of population bottlenecks. Tests were done using the approaches described by Cornuet and Luikart [35] and Garza and Williamson [36]. Under the Cornuet and Luikart method, the estimates of expected heterozygosity based on allele frequencies (*H*_*E*_), and on the number of alleles and sample size (*H*_*EQ*_) were compared, based on the assumption that the allele diversity is reduced faster than the heterozygosity. The estimates were calculated under the infinite allele model (IAM) and stepwise-mutation model (SMM) with 1000 iterations, and the allele frequency graphical mode. Significance was tested using the Wilcoxon test with the Bonferroni correction, all implemented in BOTTLENECK v1.2.02 [35]. Under the Garza and Williamson method [36], the mean ratio of the total number of alleles (*k*) to the range in allele size (*r*) is calculated (*M-ratio*), where *k* is reduced faster than *r*. Thus, a reduced *M-ratio* indicates a population having been through a bottleneck. The significance of the ratio was evaluated using the critical value (*Mc*) under the two-phase mutation model (TPM), with the average size of non one-step mutations = 2.8, as determined by [36], and θ values from 0.01, 10, and 50. Mean *M-ratio* and *Mc* were estimated using M_P_VAL and Critical_M software, respectively [36].

#### Chloroplast diversity

Genetic variation was evaluated for each population and region. The variation was given by number of polymorphic sites (*S*), number of haplotypes (*h*), haplotype diversity (*H*d), average per site pairwise nucleotide diversity (π), and the relationship between polymorphic sites and alleles sampled (θw). The parameters were estimated using DnaSP v5.10.01 [37].

#### Genetic differentiation in nuclear microsatellite data

Significant genetic structure was determined using two different population-based measures. Pairwise genetic differentiation analysis was performed using *F*_ST_ [38] and *D*est [39] with *a priori* groups of individuals according to their geographic position. The *F* estimator of genetic structure *θ* per locus and sample was calculated using an analysis of variance (FSTAT v2.9.3.2) [40], where the populations are weighted according to their sample size [38].

Patterns of structure were also assessed by permuting allele sizes among alleles and the calculation of the *F*_ST_ and the *R*_ST_. Permuted values of *R*_ST_ larger than *F*_ST_ indicate a contribution of stepwise mutations to differentiation among populations. Tests were performed in SPAGeDi v1.3 [41] using 10,000 permutations under a regression analysis restricted to population level. *R*_ST_ was used to account for the mutational distances among alleles under the stepwise mutation model [42]. *R*_ST_ is analogous to *F*_ST_, but it is based on allele size rather than allele identity.

A genetic differentiation estimate was calculated. The estimator *D*est [39] was implemented in the application SMOGD using 1000 bootstrap replicates [43]. *D*est is determined by mutation and migration rates and number of populations, and it is independent of population size [44]. It is more suitable for describing allelic differentiation among populations than other estimates such as *G*_ST_.

To detect the number of genetically differentiated populations without *a priori* assumptions of population subdivision, a Bayesian clustering analysis was used. The analysis was run under an admixture model with correlated allele frequencies for 10 million iterations after a burn-in period of 100,000 iterations for each value of *K,* and 10 replicates per run. Population structure was inferred using a range of *K* from one to six. The analysis was performed in STRUCTURE v2.3.3 [45]. The Evanno method [46] was implemented in STRUCTURE HARVESTER [47] to estimate the most likely *K* based on the likelihood scores. The analysis estimates coancestry coefficients for individuals assigned to each of *K* populations.

#### Genetic differentiation in chloroplast sequence data

A spatial analysis of molecular variance (SAMOVA) was conducted to identify groups of related populations based on geographical and genetic distances [48]. This approach, which does not consider *a priori* structure parameters, identifies groups that are geographically homogeneous and genetically differentiated. The most likely number of groups (*K*) was determined using 10,000 iterations and 10 repetitions for *K* values from two to eight groups. The most likely structure was defined by the maximum value of Φ_CT_ (the proportion of genetic variation among groups of populations) that did not include any groups of a single population [49]. The analyses were carried out using SPADS v1.0 [50].

The relationships among chloroplast haplotypes were also assessed using statistical-parsimony networks implemented in R Statistical Software with the package PEGAS [51, 52].

#### Spatial genetic patterns

The genetic structure was further analyzed with the Monmonier maximum difference algorithm to confirm the significance of structure recovered with previous metrics. The algorithm was used to locate areas of maximum genetic distance within a geometric network of geo-referenced points constructed with a Delauney triangulation.

Two different bootstrapped genetic distance matrices were used, one using nuclear microsatellite data, *D*est [53], and other using chloroplast sequence data, Φ_ST_ [49]. Matrices were bootstrapped to generate 100 datasets. A multilocus weighted average Φ_ST_ estimator was computed using SPADS v1.0 [50] and visualized in BARRIER v2.2 [54] to generate a graphical output (Voronoï polygonation). The number of barrier segments analyzed ranged from two to eight. The number of overlapping segments between marker types was determined as well as those segments unique for a marker.

#### Phylogenetic relationships and divergence of haplotypes lineages

The relationships among chloroplast haplotypes were assessed with a phylogenetic analysis based on Bayesian inference in BEAST v1.7.5 [55]. The chloroplast dataset was concatenated to derive alleles and consisted of 34 haplotypes identified previously using the software DnaSP. Outgroup sequences were generated for *Dipteronia sinensis*, and *Acer glabrum* var. *neomexicanum* and downloaded from GenBank for *A. buergerianum* subsp. *ningpoense* (KF753631.1). *Dipteronia* and *Acer* are sister clades (subfamily Hippocastanoideae) [56]. The best-fit nucleotide substitution model was estimated using jModeltest v2.3 [57, 58]. The GTR nucleotide substitution model was chosen under an Akaike Information Criterion (AIC). Uncorrelated lognormal relaxed molecular clocks with a coalescent approach prior assuming constant population size were used. For divergence analyses, a lognormal prior was placed on the tree root, using substitution rates for the chloroplast regions that were calculated using a pairwise sequence divergence analysis (*Acer-Dipteronia*) with Jukes-Cantor correction. The resulting estimate (*Dxy* = 0.06788, SD 0.04) was divided by twice the divergence time estimated for *Acer* (14.6 Ma, [51]) to obtain per-lineage rate subs/site/Myr (0.06788/29.2 = 0.0023/1000000). The analysis comprised two independent runs with 10^7^generations, sampling every 1,000 generations and discarded the first 25 % as burn-in. Effective sample sizes (ESS) of parameter estimates and convergence and mixing of runs were assessed using Tracer v1.5 [59]. The results of separated runs were combined using LogCombiner v1.7.5 [55]. A maximum clade credibility tree was generated in TreeAnnotator v1.7.5 [55] and examined with FigTree v1.7.5 [55].

#### Hypothesis testing: single and multiple glacial refuge models

Coalescent simulations were used to test whether patterns of population differentiation are consistent with the single or multiple refuge models. The two *a priori* hypotheses of Pleistocene population structure tested were the single refugium null model and the multiple glacial refuge model. The gene-tree population-tree approach was applied, following Knowles [60] and implemented in MESQUITE v3.04 [61]. Haplotype gene trees (1000 replicates) were simulated by neutral coalescence with invariable effective population size (*Ne*) until total coalescence. Five arbitrary *Ne* were tested (10,000, 50,000, 100,000, 150,000, 225,000). The amount of discordance between the gene trees and the hypothesized populations trees was measured by the *s* statistic [62]. The *s* value for the haplotype tree was first obtained and then, the distribution of simulated *s* values for each population tree was analyzed and graphed.

The single refugium hypothesis assumes that extant populations of sugar maple are derived from a refugial population, with expansion beginning as the glaciers retreat. The divergence time of populations was estimated to early, middle and most recent Pleistocene glacial events. The null model of fragmentation of a single ancestral population is rejected if the *s* value for the haplotype tree falls outside of the 95 % confidence interval for that model [61].

Under the multiple glacial refuge model, the extant population structure of haplotypes results from isolation and divergence within four hypothesized glacial refugia. Glacial refugia were represented by assemblages of populations from: a) West Mexico, b) Midwest USA c) Southeast USA – East Mexico – Guatemala, d) Northeast USA. We tested whether populations a and b were isolated since early Pleistocene (30,000 generations / 1.2 Ma) or before (98,750 generations / 3.9 Ma); and a recent split of c and d groups at 6250 generations / 250,000 ybp, or 3500 generations / 140,000 ybp or 500 generations / 20,000 ybp.

## Results

### Nuclear microsatellite diversity

A total of 17 alleles were detected at the six microsatellite loci. The total number of observed alleles varied from two (MAP40) to seven (MAP34), and allelic richness ranged from 1.5 to 2.1. Private alleles were present in one locus (MAP34) in the Tamaulipas, Chiapas, Jalisco, and Maine populations. Departures from the HWE were found in two populations from western Mexico and four from the USA (midwestern: 2, northeastern: 1, and southeastern: 1) (Table 1). Observed heterozygosity ranged from 0.133 ± 0.1 to 0.340 ± 0.1 and expected heterozygosity from 0.132 ± 0.08 to 0.284 ± 0.1 (Table 1). The *F*_IS_ per population ranged from -0.47 to 0.395. Two populations in Mexico showed a tendency toward increased homozygosity (Guerrero 0.005, Chiapas 0.09), whereas among the USA sites, only populations from Vermont, Ohio, and Tennessee did not exhibit this homozygosity pattern (Table 1). *F*_IS_ significantly differed between the tropical peripheral sites (Chiapas–Guatemala) and the populations in USA (southeastern *U* = 12, *P* = 0.039; midwestern *U* = 14.5, *P* = 0.02; northeastern *U* = 22.5, *P* = 0.04).

**Table 1:** Diversity values obtained from nuclear data (microsatellites)^1^

Locality	HWE, P-Values	Mean number of alleles	Mean H_O_ (± SE)	Mean H_E_ (±SE)	*F*_IS_	Bottleneck graphical shape	Bottleneck under IAM (*P*)	Bottleneck under SMM (*P*)	Mean *M* value	*Mc*
Maine, U.S.A.	0.06	1.833	0.222 ± 0.14	0.263 ± 0.12	0.154	Shifted mode	0.0625	0.0625	0.4962	0.4445
Vermont, U.S.A.	0.199	1.667	0.150 ± 0.11	0.122 ± 0.08	−0.227	Normal L-shaped distribution	0.9375	0.9375	0.4648	0.4630
Pennsylvania, U.S.A.	0.013	1.833	0.270 ± 0.16	0.284 ± 0.10	0.047	Shifted mode	0.09375	0.09375	0.5204	0.4630
Ohio, U.S.A.	0.723	1.833	0.219 ± 0.14	0.170 ± 0.10	−0.283	Normal L-shaped distribution	0.875	1	0.4684	0.4630
Michigan, U.S.A.	0.033	1.667	0.133 ± 0.11	0.220 ± 0.11	0.395	Shifted mode	0.125	0.8125	0.4926	0.4630
Illinois, U.S.A.	0.011	1.833	0.183 ± 0.14	0.232 ± 0.10	0.213	Normal L-shaped distribution	0.5625	0.90625	0.5204	0.4445
Tennessee, U.S.A.	0.632	2	0.250 ± 0.13	0.247 ± 0.1	−0.011	Shifted mode	0.4375	0.84375	0.5239	0.4630
Alabama, U.S.A.	0.019	1.5	0.133 ± 0.13	0.152 ± 0.08	0.122	Shifted mode	0.8125	0.8125	0.4891	0.4630
Tamaulipas, Mexico	0.109	2.164	0.3 ± 0.14	0.225 ± 0.10	−0.331	Normal L-shaped distribution	0.125	0.8125	*0.4997*	*0.6359*
Ojo de Agua del Cuervo, Jalisco, Mexico	0.005	1.667	0.165 ± 0.12	0.132 ± 0.08	−0.247	Normal L-shaped distribution	0.8125	0.9375	*0.4648*	*0.6388*
Sierra de Manantlan Biosphere Reserve, Jalisco, Mexico	0.015	1.833	0.196 ± 0.13	0.147 ± 0.08	−0.33	Normal L-shaped distribution	0.8125	0.9375	*0.4926*	*0.5984*
Guerrero, Mexico	0.151	2	0.214 ± 0.12	0.215 ± 0.08	0.005	Normal L-shaped distribution	0.90625	0.9375	0.5482	0.5085
Chiapas, Mexico	0.452	2.165	0.222 ± 0.11	0.244 ± 0.09	0.09	Shifted mode	0.84375	0.96875	0.5275	0.4445
Quiche, Guatemala	0.204	1.667	0.294 ± 0.14	0.210 ± 0.09	−0.401	Shifted mode	0.125	0.1875	*0.4891*	*0.5356*
Zacapa, Guatemala	0.141	2	0.340 ± 0.15	0.256 ± 0.11	−0.331	Shifted mode	0.0625	0.125	*0.4962*	*0.6061*
El Progreso, Guatemala	0.208	1.833	0.281 ± 0.16	0.191 ± 0.10	−0.47	Normal L-shaped distribution	0.8125	0.9375	0.4697	0.4630

^1^Hardy-Weinberg Equilibrium (HWE); Observed Heterozygosity (H_*O*_), Expected Heterozygosity (H_*E*_), Inbreeding coefficient (*F*_IS_). Bottleneck estimates under the Stepwise Mutation Model (SMM), the Infinite Allele Model (IAM), as well as the M ratio test using θ = 50. M ratio below *Mc* indicates a bottleneck (in italics)

Genetic signatures of population bottlenecks were detected, although there was disagreement between the different tests used. The *M ratio* test indicated that only populations located in the northern and western Mexico and Guatemala experienced a bottleneck, likely lasting several generations. The Wilcoxon test did not indicate a significant recent bottleneck (heterozygosity excess) in any of the populations and under any models. These results were not consistent, however, with the distribution of allele frequency. Eight populations (one in Mexico and two in Guatemala) displayed a mode shift, indicating that a bottleneck had occurred. In contrast, the normal L-shape distribution of allele frequencies indicated that eight populations had not experienced a recent bottleneck. Under the mutation-drift equilibrium scenario, the rarest allele class was more frequent, forming an L-shape graph [63]. After a bottleneck, the rarest alleles were rapidly lost, resulting in a mode-shift distortion (Table 1).

### Chloroplast diversity

A number of haplotypes were recovered from two chloroplast regions. Chloroplast sequences of the *psbJ-petA* and the *ndhF-rpl32R* intergenic spacer were 729 and 770 nucleotides long, respectively*.* The total number of haplotypes in the concatenated sequences was 34, and diversity *H*d = 0.8609. The spacer *ndhF-rpl32R* had a haplotype diversity *H*d = 0.667, and nucleotide diversity π = 0.00295 [53]; and the spacer *psbJ-petA* had *H*d = 0.620 and π = 0.00168.

Haplotype diversity was variable among populations (*H*d from 0-1). A site in Guatemala had only one haplotype, whereas larger numbers of haplotypes occurred in the southeastern and midwestern USA (Table 2). Nucleotide diversity was low in all populations (π from 0–0.00288), but the southeastern USA region had the highest (π = 0.00253), followed by the midwestern region (π = 0.00109). The site with the lowest nucleotide diversity was in western Mexico (π = 0.0009) (Table 2).

**Table 2:** Nucleotide polymorphism and diversity in *psbJ-petA* and *ndhF-rpl32R* chloroplast regions^2^. Values are given by population and by regions

Locality	(S)	(h)	(Hd) (±1 SD)	(π) (±1 SD)	(qw)
Maine, U.S.A.	3	4	0.81 ± 0.13	0.00072 ± 0.00019	0.00084
Vermont, U.S.A.	1	2	0.4 ± 0.24	0.00028 ± 0.00016	0.00033
Pennsylvania, U.S.A.	4	5	0.786 ± 0.15	0.00082 ± 0.00023	0.00107
Ohio, U.S.A.	5	4	0.643 ± 0.18	0.00098 ± 0.00045	0.00133
Michigan, U.S.A.	1	2	0.476 ± 0.17	0.00033 ± 0.00012	0.00028
Illinois, U.S.A.	8	5	0.857 ± 0.14	0.0017 ± 0.0006	0.00225
Tennessee, U.S.A.	12	7	1 ± 0.07	0.00288 ± 0.00092	0.0034
Alabama, U.S.A.	2	2	1 ± 0.5	0.00138 ± 0.00069	0.00138
Tamaulipas, Mexico	7	3	0.378 ± 0.18	0.00096 ± 0.00063	0.00171
Ojo de Agua del Cuervo, Jalisco, Mexico	4	4	0.643 ± 0.18	0.00069 ± 0.00027	0.00106
Sierra de Manantlan Biosphere Reserve, Jalisco, Mexico	2	2	0.25 ± 0.18	0.00035 ± 0.00025	0.00053
Guerrero, Mexico	1	2	0.667 ± 0.31	0.00046 ± 0.00022	0.00046
Chiapas, Mexico	5	4	0.8 ± 0.17	0.00129 ±0.00046	0.00151
Quiche, Guatemala	3	4	0.694 ± 0.15	0.00058 ± 0.00017	0.00076
Zacapa, Guatemala	0	1	0 ± 0	0 ± 0	0
El Progreso, Guatemala	3	3	0.524 ± 0.20	0.00059 ± 0.00028	0.00084
Regions					
Northeast USA	5	6	0.732 ± 0.1	0.00071 ± 0.00015	0.00101
Midwest USA	8	6	0.762 ± 0.08	0.00109 ± 0.00038	0.00169
Southeast USA	13	8	0.972 ± 0.06	0.00253 ± 0.00081	0.00332
Western MX (Jalisco and Guerrero states)	9	7	0.608 ± 0.13	0.0009 ± 0.00025	0.00178
South MX and Guatemala	10	8	0.484 ± 0.11	0.00055 ± 0.00017	0.00173

^2^Number of polymorphic sites (S), number of haplotypes (h), haplotype diversity (Hd), average per site pairwise nucleotide diversity (π), relationship between polymorphic sites and alleles samples (qw)

### Genetic differentiation in nuclear microsatellite data

There was variation in genetic structure across the 16 populations. The pairwise θ (*F*_ST_) values ranged from -0.0703 to 0.5082 indicating differentiation among populations (Additional file 2). The values for *D*est were low and varied from 0.00008 to 0.0911 (Additional file 2). A permutation test of pairwise *R*_ST_ and *F*_ST_ indicated that alleles were more related between nearby populations than between distant populations (slope coefficient *b* = 0.0075).

The results given by the estimator *D*est, which accounts for small sample sizes, indicated virtually identical allele frequencies among populations in Illinois, Michigan, and Pennsylvania, as well as between populations in northern Mexico and one in Guatemala. All values of genetic differentiation were low, with the highest differentiation occurring between the northeastern USA populations and populations in Guerrero (west) and Chiapas (south) in Mexico (Additional file 2). Populations in the midwestern USA differed from those in Guerrero and Jalisco (western Mexico), as well as from Guatemalan populations (Additional file 2). Consistent with the *D*est estimator, the highest differentiation, given by the *F*_ST_ metric, occurred between northeastern USA and the Mexican populations from Guerrero, Jalisco (west), and Chiapas (south) (Additional file 2).

The Bayesian clustering analysis was moderately informative. The highest log probability of the data, Ln *P(D)* = -1158.03, inferred that the most likely number of clusters (*K*) was two (Additional file 3). Membership coefficients for one cluster ranged from 0.617 to 0.816 and from 0.723 to 0.934 in the second cluster.

### Genetic differentiation in chloroplast sequence data

The optimal number of groups indicated by SAMOVA was *K* = 4 (Φ_CT_ = 0.545 *P* = 0.001; 0 singleton populations). Higher levels of *K* included single-population groups with only a slight increase in the index (Additional file 4). The genetic structure identified by SAMOVA (*K* = 4) consisted of one group composed by the populations from Guatemala, Mexico (excluding Jalisco), the Southeast USA, and Ohio. A second group included only the populations from western Mexico (Jalisco), and the Northeast and the Midwest USA populations each composed the third and fourth group.

The number of chloroplast haplotypes from all *Acer* populations was 34. Haplotype “AA” was the most frequent and widespread, occurring in half of the sites (Guatemala, northern and southern Mexico, southeastern USA, and Ohio) (Fig. 1). The second most frequent were the haplotypes “H” and “Y”, both with the same frequency. Haplotype “Y” was exclusive to northeastern USA, whereas “H” was unique to populations in western Mexico. Haplotypes “M” and “K” were frequent in the midwestern USA sites (Fig. 1). “E” and “F” were exclusive to western Mexico and “D” to northeastern USA. The remaining haplotypes were unique and confined to a single region.

### Spatial genetic patterns

Geographic boundaries detected by the Monmonier algorithm supported the separation of midwestern USA and western Mexican sites. The bootstrap support of the segments ranged from 46–55 % (Fig. 1), whereas SAMOVA results, based on chloroplast data, supported the regional subdivisions (Φ_CT_ = 0.545 *P* = 0.001).

Boundaries were also indicated using nuclear data. Strong genetic change occurred among populations in Mexico and USA and between Mexican and Guatemalan populations, suggesting current limits to gene flow (bootstrap support 41–58 %) (Fig. 1). In addition, results indicated a higher subdivision in those populations in tropical regions (Fig. 1).

### Phylogenetic relationships and divergence of haplotypes lineages

**Fig. 2:**
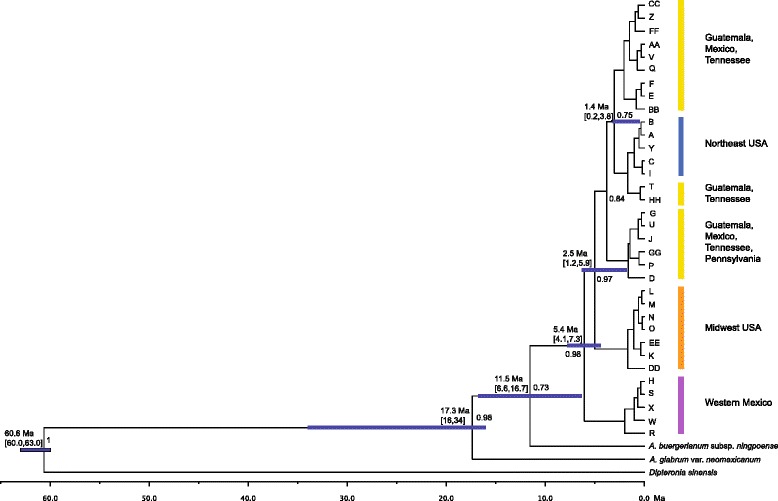
Maximum clade credibility tree of chloroplast haplotypes showing phylogenetic relationships among samples of sugar maples occurring in North and Central America*.* Posterior probabilities and mean divergence time for haplotype lineages are given. Numbers in brackets indicate the highest 95 % posterior density intervals. Colors correspond to the haplotypes depicted in Figure 1

The Bayesian topology resolved three main and well-supported clades. Relationships among the haplotypes from western Mexico (Jalisco) received high support (0.98) (Fig. 2). Haplotypes from the Midwest USA formed a well-supported monophyletic group with high posterior probability (0.98) (Fig. 2). The rest of the haplotypes formed a group with a probability of 0.84. Haplotypes from Guatemala, Mexico, and Tennessee were related to those from the Northeast USA (Fig. 2). Divergence dating for haplotypes estimated a time for the most common ancestor between the lineage occurring in Jalisco (western Mexico) and the rest of the tropical and temperate sugar maple phylogroups as the Pliocene, at 5.4 Ma (95 % HPD: 4.1–7.3). A split between the haplotypes from the midwestern USA and the phylogroups from Mexico, Guatemala, southeastern USA, and northeastern USA was dated to the early Pleistocene, at 2.5 Ma (95 % HPD: 1.2–5.9), whereas the Northeast USA lineage split was in the Pleistocene, at 1.4 Ma (95 % HPD: 0.2–3.8) (Fig. 2).

### Hypothesis testing: single and multiple glacial refuge models

The single refugium null model was not supported by the data. The Slatkin and Maddison’s *s* statistic from the gene tree was *s* = 14. The observed value of *s* = 14 was not within the 95 % confidence interval of the simulated distribution under any of the different times of population splitting and for all *Ne* values.

The multiple glacial refuge model could not be rejected. The observed *s* = 14 falls within the 95 % confidence interval (Additional file 5). The simulations for the estimate of *Ne* = 225,000 with a Southeast – Northeast USA population divergence at 250,000 ybp and population split at 1.2 Ma of the Midwest USA group were supported.

## Discussion

Data on nuclear and chloroplast DNA of sugar maple collected from North and Central America recovered signatures of past connectivity, disjunctions, and genetic structure among populations. Populations in western Mexico (Jalisco) and the midwestern USA had the highest genetic differentiation; their haplotype lineages diverged, respectively, during the Pliocene and early Pleistocene. Further differentiation of northeastern USA haplotype lineages was dated to the pre-Illinoian glacial episode (Pleistocene). Population in the southeastern USA, eastern Mexico, and Guatemala displayed signals of past connectivity and lack genetic differentiation. In addition, most populations in the tropics had increased homozygosity and lower genetic diversity, possible as a result of bottlenecks lasting many generations. Haplotype data suggested the presence of multiple glacial refugia; the tropical populations likely acted as refugia during the Quaternary glacial and interglacial events, while two other refugia were maintained in the Midwest and north of the Appalachian Mountains.

### Genetic diversity and geographic structure

Low levels of genetic diversity from nuclear DNA data were observed in all populations. A greater tendency toward homozygosity occurs in maples from western and southern Mexico compared to maples Vermont, Ohio, and Tennessee. The trend could be the result of a genetic bottleneck in southern Mexico. The reduced genetic variation is also present in the Guatemalan populations. The increased homozygosity in southern tropical populations indicates that populations at the most southern ends of the range of this species should be the most vulnerable to new selection pressures. Populations in southern Mexico and Guatemala are also the most threatened by anthropogenic disturbance [19]. Consistently, low values in nuclear markers with high mutation rates reflect fragmentation patterns and reductions in population sizes in recent times. Low to moderate diversity has also been observed in *Acer saccharum* in populations from Canada, possibly a result of genetic drift [23–25]. Nevertheless, genetic variation may be underestimated across species using the same markers, because microsatellites are subject to ascertainment bias [64]. In addition, a more accurate estimation of genetic diversity should be obtained by increasing the number of microsatellites.

Chloroplast gene diversity in most maple populations was variable. High levels of nucleotide and haplotype diversity were distributed in the southern and the midwestern regions of the USA. High nucleotide diversity (π) also was also observed in the southern population of Chiapas, Mexico. Nevertheless, populations in tropical regions contained the lowest haplotype diversity. Gunter *et al*. [65] found the greatest sugar maple genetic diversity in the southern region of Tennessee and the lowest in the northern area of Wisconsin. Latitude was suggested to be responsible for trends in genetic diversity [66]; no significant latitudinal pattern in genetic diversity was observed, however, when analyzing the chloroplast DNA [26]. Rates of chloroplast evolution are slow compared to nuclear microsatellites; thus, the chloroplast data could reflect past population sizes and past gene flow between southeastern USA and the easternmost tropical maples of Mexico and Guatemala.

The genetic structure recovered with nuclear and chloroplast DNA indicates general patterns of connectivity among populations. Nuclear data suggest current gene flow and larger effective population sizes in southeastern and northeast USA, whereas the opposite is true in the tropical populations, having disjunct and geographically fragmented populations. Chloroplast data indicate that very little gene flow has occurred among the midwestern sites and the rest of the populations in the USA since some distant time in the past.

Haplotypes from more southern regions may be considered ancestral, which is suggested by high frequency and distribution of haplotypes in southeastern USA and south Mexico and Guatemala. Haplotype lineages in western Mexico (Jalisco) were the first to diverge, during the Pliocene. Eastern Mexican and Guatemalan lineages had a time for the most common ancestor similar to the northeastern USA populations, dated to the pre-Illinoian glacial period. The haplotypes from the Midwest, supported as a lineage by Bayesian inference, have survived isolated since the early Pleistocene (when ice-sheets began to grow), with possible gene migrations from more central (Ohio) or southern (Alabama) ice-free areas. Samples from Iowa, Missouri, as well as sequences of other chloroplast regions, may help to clarify the routes of migration for the midwestern populations.

### Patterns of genetic differentiation

The results from the STRUCTURE analysis suggested that the genotyped individuals fall into two general clusters. Thus, there is one cluster from the tropics, which also includes Alabama, and one cluster composed of USA sites, but excludes the Southeast USA. These results contrasted with *D*est and *F*_ST_ measures. Bayesian clustering analysis did not differentiate the western Mexico populations, whereas *D*est and *F*_ST_ gave strong support for these populations as a different group. Similarly, the population in Chiapas was assigned to the USA cluster with STRUCTURE, but it was differentiated using *D*est and *F*_ST_. The Chiapas population might represent an outlier, possible due to the high levels of homozygosity and reduced population sizes. Caution is needed in making inferences, however, because the accuracy of the assignments in STRUCTURE is affected when there are insufficient microsatellite markers [45].

Populations in midwestern USA and western Mexico were highly differentiated from the rest of the sites under study. *D*est and *F*_ST_ supported differentiation for nuclear DNA and SAMOVA for chloroplast DNA. Their isolation thus appears to be old and is supported by the time for the most common ancestor estimates for haplotypes in this study. Our data further suggest very limited present gene flow (via pollen dispersal) from Alabama to the populations in the Midwest.

The nuclear DNA also indicates lack of gene flow at the present time between the northern Mexico and southern USA populations. Nonetheless, the signal of past connectivity among the Southeast USA, East Mexico, and Guatemalan populations is still present, as indicated by the low chloroplast differentiation among these populations. Similar signals of past connectivity have been identified between populations of *Liquidambar styraciflua* in the southeastern USA and Mexico [8]. Populations in Mexico also exhibit differentiation from each other as a result of geographic barriers and a highly fragmented landscape. Although geographically close, Guatemalan populations also displayed a lack of pollen flow. This suggests that in addition to a mountain barrier, the population sizes are small, pollen dispersion is limited, and there are no intermediate patches of maples that might increase connectivity at the present time. Similar degrees of reproductive isolation after the last glaciation and landscape fragmentation have been recorded for other temperate trees such as *Fagus grandifolia* and *Pinus chiapensis* in Mexico [13, 15].

### Pliocene–Quaternary glacial and interglacial effects on populations

#### Pliocene events and western Mexico population divergence

Populations of *Acer saccharum* could have become established in western Mexico during the Pliocene or late Miocene, when the temperatures were low. The presence of other temperate tree genera (e.g., *Platanus* and *Populus*) during the Pliocene in central Mexico is supported by the presence of macrofossils [67]. In addition, a probable arrival in Mexico during the Miocene/Pliocene has been suggested for other cloud forest trees, such as *Liquidambar* and *Podocarpus matudae* [68–70].

The uplift of the Trans-Mexico Volcanic Belt, a major mountain barrier stretching from west to east across the country, isolated western areas of Mexico in the late Miocene. This mountain belt was characterized by major volcanic activity during the late Pliocene and the Quaternary, mainly along the western side [71, 72]. The volcanic activity and extensive accumulation of volcaniclastic particles during the Quaternary could have isolated western maple populations in Jalisco and Guerrero by preventing immigration, thereby facilitating the divergence of already established populations. Further, the volcanic belt could have blocked northern cold fronts, creating climatic conditions that promoted growth of ample areas of dry forests in western Mexico as well as fragmenting and reducing cold and humid areas, which are most suitable for maple establishment [73]. This ancient division among maples is suggested by the high differentiation between western populations and the rest of the study sites, the presence of different haplotypes, and strong barriers, as well as by the time for the most common ancestor values. These findings suggest that the taxonomic status of the western Mexico populations needs to be reconsidered (Vargas-Rodriguez in review).

#### Pleistocene effects on populations

Glacial periods during the Pleistocene could have favored species expansions with continuous gene flow through eastern–southern Mexico to Guatemala, which then ended after the last glacial maximum (18,000 years B.P.). Pollen from cores of the Basin of Mexico indicates three glacial advances during 30,000–25,000 years B.P., 12,000 years B.P., and during late Holocene [74, 75]. A relative stable humidity during glacial events could have facilitated expansion and population connectivity in the tropical areas. A warm period of the middle Holocene and the “Medieval Warm Period” (1038–963 years B.P.) [76] might have helped produce the current fragmented distribution of *Acer* in cloud forests. Even though the Trans-Mexico Volcanic Belt reaches eastern Mexico, it had reduced volcanic activity along its eastern side [71] and, together with the presence of continuous mountain ridges, this might have allowed a steady connection among populations in eastern–southern Mexico and Guatemala. The sites in Ohio and southern USA share the same chloroplast haplotypes with those in northern and southern Mexico and Guatemala sites, but they are differentiated from the Jalisco and, to a lesser extent, the Guerrero populations (western Mexico).

#### Hypotheses of Pleistocene refugia

Small maple refugia may have existed in the Midwest USA and north of the Appalachian Mountains. A population in the Midwest might have persisted through the glaciations from the beginning of Pleistocene, experienced possible gene flow from the sites in the south (Alabama), but remained isolated from the more eastern sites (Ohio, Tennessee). The Mississippi River Valley is hypothesized to have increased genetic differentiation to the east and the west of the river in *Pinus* and *Juglans* [1], and the river valley probably also affected the migration of *Acer* during the Pleistocene. Another small maple refugium should have occurred in Ohio, based on the diversity of haplotypes. The Ohio population also shared a larger proportion of the most abundant haplotype with the southeastern and tropical sites, and a smaller proportion of haplotypes from the northeastern site. Maples in the Northeast might have expanded from a refugium in the northern area of the Appalachian Mountains. During the last glaciations, ice-free areas existed in the Pennsylvania region, from where the maples could have migrated following deglaciation [77]. The low haplotype diversity found here suggests genetic drift in small refugial populations from the Northeast. It has been proposed that plants survived ice ages on small island-like areas protruding above the ice-sheets in the North Atlantic zone; however, climatically harsh conditions also could have prevented *in situ* glacial survival of maples [20, 78, 79]. Thus, maples in the Northeast likely migrated from periglacial areas of the Pennsylvania region [80]. On the other hand, physiographic conditions of northern Appalachians restricted maple gene flow from the south to the northeast during the last 14,000–10,000 years B.P. [22]. This barrier in gene flow is supported by the haplotype data. Low-density populations of *Acer rubrum* and *Fagus grandifolia* could also have persisted in periglacial areas in Ohio, expanding following deglaciation and making a more important contribution to species expansion than other populations in the southeastern USA [9, 11, 12]. Thus, the assumption of a single refugium of sugar maples in the Gulf Coastal Plain during the glaciations in the Pleistocene does not appear to be valid [1, 17]. Our results supported that the idea that the midwestern USA has provided refugia most likely onward from the early Pleistocene. Parts of the area remained ice-free during the last glaciation and allowed the persistence of deciduous species [81]. Recent phylogeographic evidence for *Smilax* also supports the hypothesis that the Midwest served as a refugium [82]. Moreover, temperate tree populations in the tropics, usually overlooked [83], have also been an important gene reservoir, acting as refugia during the Pleistocene.

#### Late Quaternary population patterns

Habitat fragmentation may have resulted from increased dry and warm conditions that followed the last glacial maximum. In addition, anthropogenic habitat transformation has affected the cloud forests containing sugar maples in Mexico and Guatemala [19]. Reduced population sizes, lack of gene flow, genetic bottlenecks, and increases in homozygosity now characterizing these populations will probably be rapidly exacerbated by ongoing anthropogenic activity. Thus, historical processes and disturbances are contracting the most southern end of the ranges of sugar maple trees. Recent strong warming conditions are promoting a northward migration of sugar maples shifting the range towards a more northern limit in Canada [84].

## Conclusions

Reduced genetic variation currently is present in the most southern tropical maple populations, as indicated by nuclear and chloroplast data. Haplotype diversity and distribution indicate past connectivity among populations from the Southeast USA, East Mexico, and Guatemala. Thus, gene flow and species expansion through eastern–southern Mexico to Guatemala was likely favored during glacial periods of stable humidity during the Quaternary. Earlier, western Mexican (Jalisco) populations diverged from the rest of the sugar maples during the Pliocene. Volcanic activity during late Pliocene and Quaternary and topographic conditions in western Mexico probably promoted this early differentiation. The time for the most common ancestor estimates denote that midwestern USA populations have been different lineages since the early Pleistocene and that northeastern USA lineages diverged during the pre-Illinoian glaciation, long before the Last Glacial Maximum. Small maple refugia may have existed in the midwestern USA and north of the Appalachian Mountains, and expansion may have occurred from such refuges during interglacial periods. Thus, we suggest that geological events in the Pliocene were major determinants of the current genetic structure of sugar maple populations in the area of West Mexico, whereas the geological events that occurred during the Quaternary explain the genetic structure of sugar maples in East Mexico and the USA. This study supports the notion of multiple glacial refugia for temperate hardwood forests in North and Central America and highlights the importance of connectivity among temperate forests in the USA and those in Central America.

## Availability of supporting data

The chloroplast DNA data set supporting the results of this article is available in GenBank, with the accession numbers KT933356–KT933397.

## Additional files

Additional file 1:
**The sugar maple populations studied and the geographic information from the sampled localities.** Population and ecological characteristics for Mexican and Guatemalan populations are given elsewhere [19]. (DOC 47 kb) Additional file 2:
**Below diagonal, FST: the proportion of genetic diversity due to allele frequency differences among populations.** Above diagonal, Dest: estimator of actual differentiation. High values are in bold and imply a degree of differentiation among populations. (DOC 49 Kb) Additional file 3:
**Graphical summary of Bayesian clustering. a)** Geographical subdivision as inferred using STRUCTURE. Two clusters are distinguishable: one (in red) includes populations from Guatemala (PR, ZA, QC), Mexico (Tamps, Tal, Man), and Alabama (AL). Membership coefficients for the red cluster ranged from 0.617 to 0.816. A second cluster in green comprises populations from Northeast USA (ME, PA), Midwest USA (MI, IL), and the most southern site in Mexico (Chiapas, Chis). Coefficients ranged from 0.723 to 0.934 in green cluster. **b)** Likelihood score differences (DeltaK) with highest difference between K = 2 and K = 3. (DOC 629 kb) Additional file 4:
**Fixation indices and number of singleton populations obtained from the spatial analyses of molecular variance (SAMOVA) of sugar maple’s chloroplast DNA data.** (DOC 28 kb) Additional file 5:
**Distribution of Slatkin and Maddison’s s statistic for 1000 simulated gene trees within a population tree.** Ne = 225,000. Observed s = 14 (asterisk) indicates that the population structure is consistent with the multiple refuge hypothesis. (DOC 219 kb)

## Abbreviations

SAMOVA: spatial analysis of molecular variance
SMOGD: software for the measurement of genetic diversity
