# Supplementary material for: Large scale patterns of genetic variation and differentiation in sugar maple from tropical Central America to temperate North America
Source: BMC Evol Biol. 2015 Nov 19;15:257. doi: 10.1186/s12862-015-0518-7 (PMC4653954; doi:10.1186/s12862-015-0518-7)
Supplement: Additional file 1: — The sugar maple populations studied and the geographic information from the sampled localities. Population and ecological characteristics for Mexican and Guatemalan populations are given elsewhere [19]. (DOC 47 kb) [file 12862_2015_518_MOESM1_ESM.doc]

Additional file 1.

The sugar maple populations studied and the geographic information from the sampled localities. Population and ecological characteristics for Mexican and Guatemalan populations are given elsewhere [19].

| Locality, arranged by latitude | State | Country | Sampled species | Latitude | Longitude |
| --- | --- | --- | --- | --- | --- |
| Big Reed Forest Reserve | Maine | U.S.A. | *A.* *saccharum* subsp. *saccharum* | 46.3581 | -69.0381 |
| Mt. Tabor, Green Mountain National Forest | Vermont | U.S.A. | *A.* *saccharum* subsp. *saccharum* | 43.3670 | -72.9668 |
| Tuscarora State Forest | Pennsylvania | U.S.A. | *A.* *saccharum* subsp. *saccharum* | 40.1327 | -77.7653 |
| Johnson Woods State Nature Preserve | Ohio | U.S.A. | *A.* *saccharum* subsp. *saccharum* | 40.8885 | -81.7440 |
| Warren Woods State Park | Michigan | U.S.A. | *A.* *saccharum* subsp. *saccharum* | 41.8373 | -86.6258 |
| Baber Woods Nature Preserve | Illinois | U.S.A. | *A.* *saccharum* subsp. *saccharum* | 39.4933 | -87.9017 |
| Smoky Mountain National Park | Tennessee | U.S.A. | *A.* *saccharum* subsp. *saccharum* | 35.7041 | -83.3312 |
| Thompson Creek, Bankhead National Forest | Alabama | U.S.A. | *A.* *saccharum* subsp. *saccharum* | 34.3407 | -87.4705 |
| El Cielo Biosphere Reserve | Tamaulipas | Mexico | *A. saccharum* subsp. *skutchii* | 23.0768 | -99.2163 |
| Ojo de Agua del Cuervo | Jalisco | Mexico | *A. saccharum* subsp. *skutchii* | 20.2123 | -104.7568 |
| Sierra de Manantlan Biosphere Reserve | Jalisco | Mexico | *A. saccharum* subsp. *skutchii* | 19.6051 | -104.2964 |
| Barranca El Silencio | Guerrero | Mexico | *A. saccharum* subsp. *skutchii* | 18.1833 | -101.4000 |
| Tenejapa | Chiapas | Mexico | *A. saccharum* subsp. *skutchii* | 16.8212 | -92.5387 |
| Nebaj | Quiche | Guatemala | *A. saccharum* subsp. *skutchii* | 15.4323 | -91.1423 |
| San Lorenzo, Sierra de las Minas Biosphere Reserve | Zacapa | Guatemala | *A. saccharum* subsp. *skutchii* | 15.0849 | -89.6762 |
| El Balsamal, Sierra de las Minas Biosphere Reserve | El Progreso | Guatemala | *A. saccharum* subsp. *skutchii* | 15.0487 | -89.9752 |
