# Supplementary material for: Large scale patterns of genetic variation and differentiation in sugar maple from tropical Central America to temperate North America
Source: BMC Evol Biol. 2015 Nov 19;15:257. doi: 10.1186/s12862-015-0518-7 (PMC4653954; doi:10.1186/s12862-015-0518-7)
Supplement: Additional file 2: — Below diagonal, FST: the proportion of genetic diversity due to allele frequency differences among populations. Above diagonal, Dest: estimator of actual differentiation. High values are in bold and imply a degree of differentiation among populations. (DOC 49 Kb) [file 12862_2015_518_MOESM2_ESM.doc]

Additional file 2.

Below diagonal, F_ST_: the proportion of genetic diversity due to allele frequency differences among populations. Above diagonal, *D*est: estimator of actual differentiation. High values are in bold and imply a degree of differentiation among populations.

|  | El Progreso | Zacapa | Quiche | Tamaulipas | Guerrero | Chiapas | Talpa Jalisco | Manantlan Jalisco | Maine | Vermont | Pennsylvania | Michigan | Illinois | Alabama | Tennessee | Ohio |
| --- | --- | --- | --- | --- | --- | --- | --- | --- | --- | --- | --- | --- | --- | --- | --- | --- |
| El Progreso |  | 0.0014 | 0.00001 | 0.0012 | 0.0175 | 0.0306 | 0.000006 | 0.0005 | 0.0327 | 0.0215 | 0.0192 | **0.0437** | 0.0294 | 0.0002 | 0.0082 | 0.0016 |
| Zacapa | 0.0444 |  | 0.0007 | 0.0005 | 0.0239 | 0.0231 | 0.0051 | 0.0040 | **0.0454** | 0.0250 | 0.0194 | **0.0600** | **0.0461** | 0.0074 | 0.0065 | 0.0100 |
| Quiche | 0.0263 | 0.0253 |  | 0 | 0.0266 | 0.0227 | 0.0060 | 0.0048 | 0.0319 | 0.0110 | 0.0061 | 0.0280 | 0.0141 | 0.0095 | 0.0006 | 0.0000008 |
| Tamaulipas | 0.0474 | 0.0163 | -0.0097 |  | 0.0290 | 0.0216 | 0.0102 | 0.0092 | **0.0403** | 0.0167 | 0.0109 | 0.0397 | 0.0244 | 0.0164 | 0.0044 | 0.0015 |
| Guerrero | **0.3517** | **0.3059** | **0.3536** | **0.3339** |  | 0.0033 | 0.0183 | 0.0186 | **0.0911** | **0.0833** | 0.0328 | **0.0434** | **0.0434** | 0.0154 | **0.0429** | 0.0373 |
| Chiapas | **0.4086** | **0.3531** | **0.3793** | **0.369** | 0.0435 |  | 0.0378 | 0.0420 | **0.0745** | **0.0586** | 0.0117 | 0.0261 | 0.0203 | **0.0439** | 0.0299 | 0.0239 |
| Talpa Jalisco | 0.0256 | 0.0825 | 0.0986 | 0.1039 | **0.3511** | **0.4611** |  | 0.0000 | 0.0387 | 0.0239 | 0.0398 | **0.0545** | **0.0454** | 0 | 0.0114 | 0.0089 |
| Manantlan Jalisco | 0.066 | 0.0685 | 0.1133 | 0.1024 | **0.3742** | **0.4767** | 0.0154 |  | **0.0449** | 0.0286 | 0.0379 | **0.0640** | **0.0517** | 0.0000 | 0.0128 | 0.0126 |
| Maine | 0.1847 | 0.1934 | 0.1817 | 0.2038 | **0.3492** | **0.3021** | **0.3318** | **0.3736** |  | 0.0118 | 0.0203 | 0.0100 | 0.0148 | 0.0362 | 0.0074 | 0.0190 |
| Vermont | 0.2285 | 0.1949 | 0.1364 | 0.1523 | **0.5082** | **0.4729** | **0.3594** | **0.4046** | 0.1066 |  | 0.0127 | 0.0052 | 0.0041 | 0.0304 | 0.0027 | 0.0017 |
| Pennsylvania | 0.152 | 0.1284 | 0.1107 | 0.1256 | 0.172 | 0.1298 | 0.2424 | 0.2673 | 0.0785 | 0.136 |  | 0 | 0 | 0.0350 | 0.00001 | 0.0006 |
| Michigan | 0.254 | 0.2375 | 0.2108 | 0.2221 | 0.2229 | 0.1306 | **0.3509** | **0.4004** | 0.0718 | 0.1614 | -0.0144 |  | 0 | **0.0502** | 0.0030 | 0.0057 |
| Illinois | 0.2133 | 0.2022 | 0.1672 | 0.1851 | 0.2102 | 0.1384 | **0.3061** | **0.3498** | 0.0694 | 0.1408 | -0.0401 | -0.0703 |  | **0.0408** | 0.0003 | 0.0015 |
| Alabama | 0.0564 | 0.0878 | 0.1351 | 0.1327 | 0.2912 | **0.3864** | -0.0032 | 0.0118 | **0.3022** | 0.413 | 0.2017 | **0.3279** | 0.2748 |  | 0.0129 | 0.0135 |
| Tennessee | 0.0617 | 0.0572 | 0.0239 | 0.0478 | **0.3205** | **0.3099** | 0.1749 | 0.2116 | 0.0511 | 0.0256 | 0.0193 | 0.0818 | 0.0485 | 0.1787 |  | 0 |
| Ohio | 0.0592 | 0.0648 | 0.0204 | 0.0424 | **0.3988** | **0.3982** | 0.1468 | 0.2014 | 0.106 | 0.0465 | 0.0822 | 0.1516 | 0.117 | 0.1893 | -0.0219 |  |
