# Supplementary material for: Large scale patterns of genetic variation and differentiation in sugar maple from tropical Central America to temperate North America
Source: BMC Evol Biol. 2015 Nov 19;15:257. doi: 10.1186/s12862-015-0518-7 (PMC4653954; doi:10.1186/s12862-015-0518-7)

Additional file 3.

Graphical summary of Bayesian clustering. a) Geographical subdivision as inferred using STRUCTURE. Two clusters are distinguishable: one (in red) includes populations from Guatemala (PR, ZA, QC), Mexico (Tamps, Tal, Man), and Alabama (AL). Membership coefficients for the red cluster ranged from 0.617 to 0.816. A second cluster in green comprises populations from Northeast USA (ME, PA), Midwest USA (MI, IL), and the most southern site in Mexico (Chiapas, Chis). Coefficients ranged from 0.723 to 0.934 in green cluster. b) Likelihood score differences (DeltaK) with highest difference between *K* = 2 and *K* = 3.


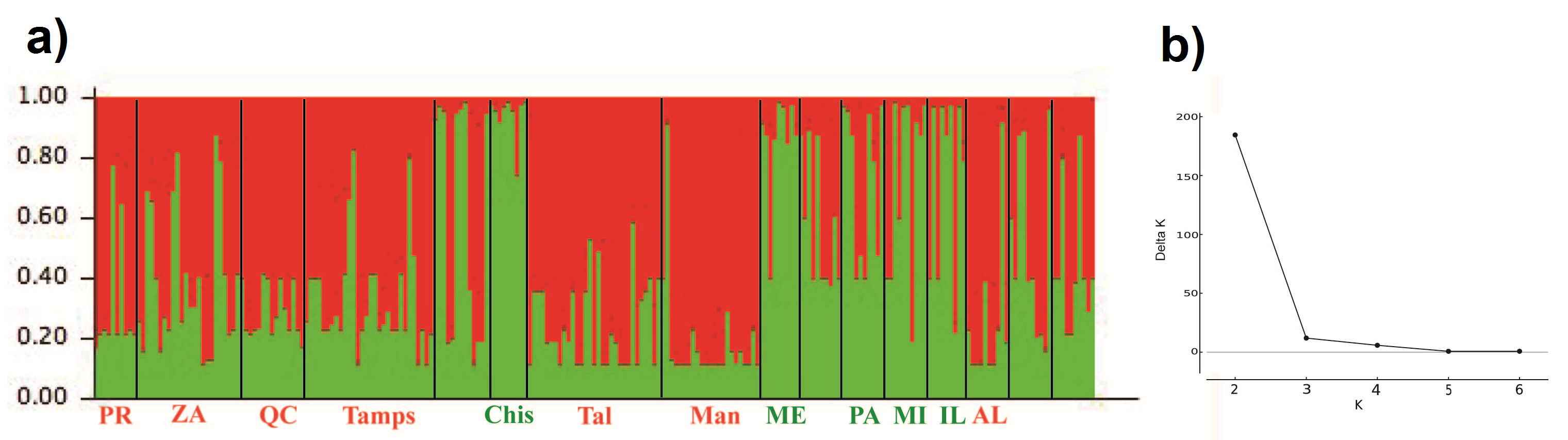

Supplement: Additional file 3: — Graphical summary of Bayesian clustering. a) Geographical subdivision as inferred using STRUCTURE. Two clusters are distinguishable: one (in red) includes populations from Guatemala (PR, ZA, QC), Mexico (Tamps, Tal, Man), and Alabama (AL). Membership coefficients for the red cluster ranged from 0.617 to 0.816. A second cluster in green comprises populations from Northeast USA (ME, PA), Midwest USA (MI, IL), and the most southern site in Mexico (Chiapas, Chis). Coefficients ranged from 0.723 to 0.934 in green cluster. b) Likelihood score differences (DeltaK) with highest difference between K = 2 and K = 3. (DOC 629 kb) [file 12862_2015_518_MOESM3_ESM.doc]
