# Supplementary material for: Large scale patterns of genetic variation and differentiation in sugar maple from tropical Central America to temperate North America
Source: BMC Evol Biol. 2015 Nov 19;15:257. doi: 10.1186/s12862-015-0518-7 (PMC4653954; doi:10.1186/s12862-015-0518-7)
Supplement: Additional file 4: — Fixation indices and number of singleton populations obtained from the spatial analyses of molecular variance (SAMOVA) of sugar maple’s chloroplast DNA data. (DOC 28 kb) [file 12862_2015_518_MOESM4_ESM.doc]

Additional file 4.

|  | Number of K-groups | Number of K-groups | Number of K-groups | Number of K-groups | Number of K-groups | Number of K-groups | Number of K-groups |
| --- | --- | --- | --- | --- | --- | --- | --- |
|  | 2 | 3 | 4 | 5 | 6 | 7 | 8 |
| Φ_CT_ | 0.51473767 | 0.4991507 | 0.5458948 | 0.5618399 | 0.553904 | 0.57607096 | 0.5702099 |
| Φ_ST_ | 0.6953689 | 0.62442696 | 0.5905582 | 0.5848126 | 0.5794371 | 0.5666655 | 0.56296253 |
| Φ_SC_ | 0.37223414 | 0.2501276 | 0.09835471 | 0.05242988 | 0.057236705 | -0.022186501 | -0.01686268 |
| Singletons | 0 | 0 | 0 | 1 | 2 | 4 | 6 |

Fixation indices and number of singleton populations obtained from the spatial analyses of molecular variance (SAMOVA) of sugar maple’s chloroplast DNA data.
