# Supplementary material for: Large scale patterns of genetic variation and differentiation in sugar maple from tropical Central America to temperate North America
Source: BMC Evol Biol. 2015 Nov 19;15:257. doi: 10.1186/s12862-015-0518-7 (PMC4653954; doi:10.1186/s12862-015-0518-7)

Additional file 5.

Distribution of Slatkin and Maddison’s *s* statistic for 1000 simulated gene trees within a population tree. *Ne* = 225,000. Observed *s* = 14 (asterisk) indicates that the population structure is consistent with the multiple refuge hypothesis.


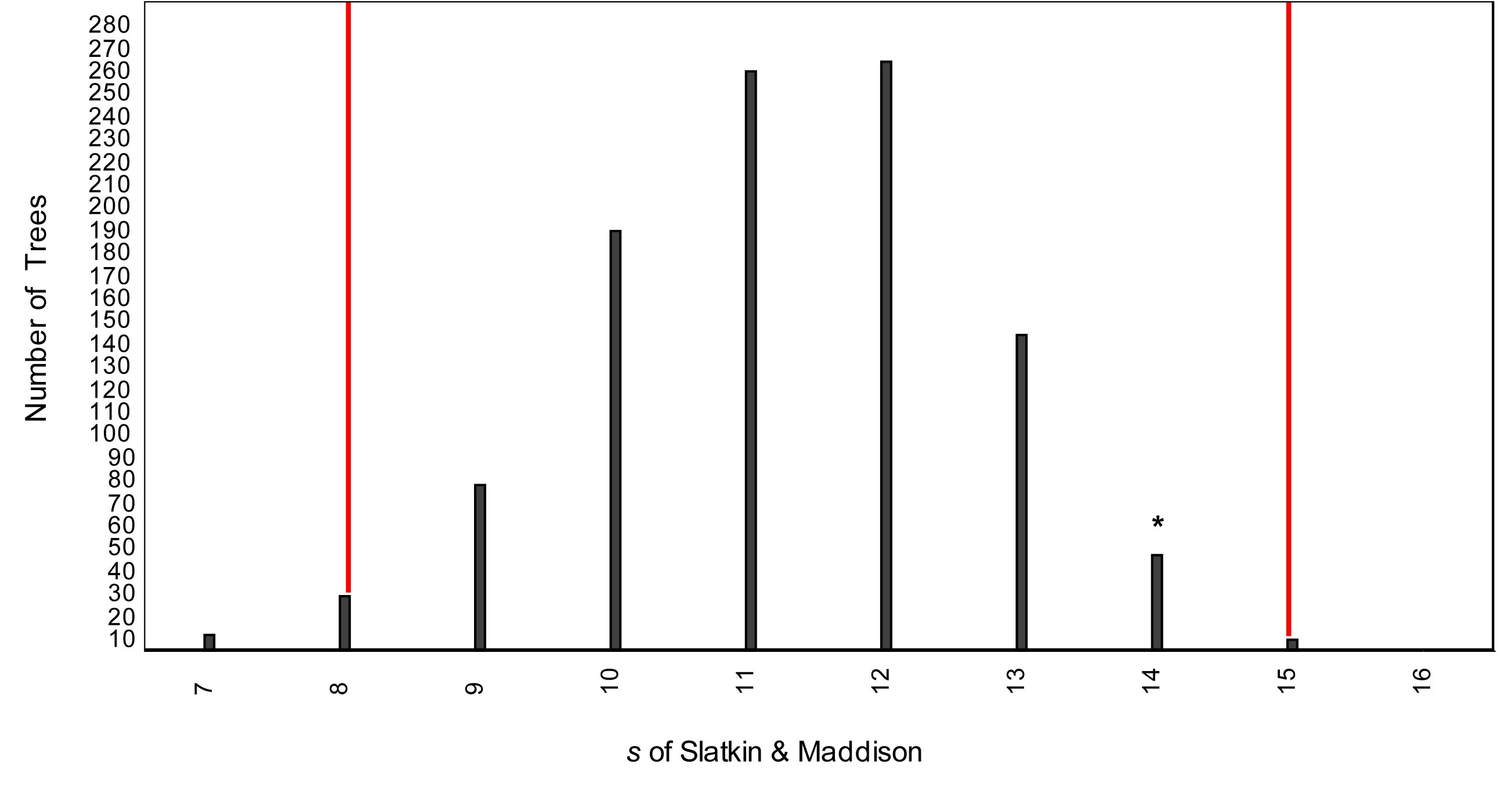

Supplement: Additional file 5: — Distribution of Slatkin and Maddison’s s statistic for 1000 simulated gene trees within a population tree. Ne = 225,000. Observed s = 14 (asterisk) indicates that the population structure is consistent with the multiple refuge hypothesis. (DOC 219 kb) [file 12862_2015_518_MOESM5_ESM.doc]
